# Pangenomic and biochemical analyses of *Helcococcus ovis* reveal widespread tetracycline resistance and a novel bacterial species, *Helcococcus bovis*

**DOI:** 10.3389/fmicb.2024.1456569

**Published:** 2024-09-10

**Authors:** Federico Cunha, Yuting Zhai, Segundo Casaro, Kristi L. Jones, Modesto Hernandez, Rafael S. Bisinotto, Subhashinie Kariyawasam, Mary B. Brown, Ashley Phillips, Kwangcheol C. Jeong, Klibs N. Galvão

**Affiliations:** ^1^Department of Large Animal Clinical Sciences, University of Florida College of Veterinary Medicine, Gainesville, FL, United States; ^2^Department of Animal Sciences, University of Florida College of Agriculture and Life Sciences, Gainesville, FL, United States; ^3^Emerging Pathogens Institute, University of Florida, Gainesville, FL, United States; ^4^Department of Comparative, Diagnostic, and Population Medicine, University of Florida College of Veterinary Medicine, Gainesville, FL, United States; ^5^D. H. Barron Reproductive and Perinatal Biology Research Program, University of Florida, Gainesville, FL, United States; ^6^Department of Infectious Diseases and Immunology, University of Florida College of Veterinary Medicine, Gainesville, FL, United States; ^7^Athens Veterinary Diagnostic Laboratory, College of Veterinary Medicine, The University of Georgia, Athens, GA, United States

**Keywords:** whole genome sequencing (WGS), *Helcococcus*, novel species, tetracycline resistance, pangenome analysis, bovine, virulence

## Abstract

*Helcococcus ovis* (*H. ovis*) is an opportunistic bacterial pathogen of a wide range of animal hosts including domestic ruminants, swine, avians, and humans. In this study, we sequenced the genomes of 35 *Helcococcus* sp. clinical isolates from the uterus of dairy cows and explored their antimicrobial resistance and biochemical phenotypes *in vitro*. Phylogenetic and average nucleotide identity analyses classified four *Helcococcus* isolates within a cryptic clade representing an undescribed species, for which we propose the name *Helcococcus bovis* sp. nov. By establishing this new species clade, we also resolve the longstanding question of the classification of the Tongji strain responsible for a confirmed human conjunctival infection. This strain did not neatly fit into *H. ovis* and is instead a member of *H. bovis*. We applied whole genome comparative analyses to explore the pangenome, resistome, virulome, and taxonomic diversity of the remaining 31 *H. ovis* isolates. An overwhelming 97% of *H. ovis* strains (30 out of 31) harbor mobile tetracycline resistance genes and displayed significantly increased minimum inhibitory concentrations of tetracyclines *in vitro*. The high prevalence of mobile tetracycline resistance genes makes *H. ovis* a significant antimicrobial resistance gene reservoir in our food chain. Finally, the phylogenetic distribution of co-occurring high-virulence determinant genes of *H. ovis* across unlinked and distant loci highlights an instance of convergent gene loss in the species. In summary, this study showed that mobile genetic element-mediated tetracycline resistance is widespread in *H. ovis*, and that there is evidence of co-occurring virulence factors across clades suggesting convergent gene loss in the species. Finally, we introduced a novel *Helcococcus* species closely related to *H. ovis*, called *H. bovis* sp. nov., which has been reported to cause infection in humans.

## Highlights

- Mobile genetic element-mediated tetracycline resistance is widespread in *H. ovis.*
- Co-occurring virulence factors across clades suggest convergent gene loss in the species.
- *Helcococcus bovis* is a novel species closely related to *Helcococcus ovis* that has been reported to cause infection in humans.

## Introduction

*Helcococcus ovis* (*H. ovis*) belongs to a clinically important genus of bacteria populated by five other described species: *Helcococcus kunzii*, *Helcococcus massiliensis*, *Helcococcus sueciensis*, *Helcococcus seattlensis*, and *Helcococcus pyogenes*, all of which are opportunistic pathogens of humans (Collins et al., 1993; Panackal et al., 2004; Chow and Clarridge, 2014; Fall et al., 2018). Unlike the remaining members of the genus, *H. ovis* is most often found as a co-infecting pathogen in mixed infections of farm animals, such as metritis (Cunha et al., 2018), mastitis (Liu et al., 2022), and pneumonia (García et al., 2012). Due to these characteristics, its ability to independently cause disease had not been documented until recently (García et al., 2012; Collins et al., 1999; Bilk et al., 2011; Locatelli et al., 2013). *H. ovis* is capable of independently causing bovine valvular endocarditis (Kutzer et al., 2009), pneumonia, and bursitis (Jost and Sickinger, 2021) in clinical infections, and mastitis in an experimentally infected mouse (*Mus musculus*) model (Liu et al., 2022). In humans, *H. ovis* DNA has been recovered from nineteen different body sites and occurs with higher relative abundance in association with peritoneal effusion, vaginal infection, and endometrial neoplasms (Jin et al., 2022). A 2008 case report details a confirmed human infection by *H. ovis* affecting ocular adnexa of an adult male working in close contact with wool and cowhide, which highlights the organism’s zoonotic potential. This then-named Tongji strain displayed an atypical biochemical profile for the species and was suggested to represent a *H. ovis* subspecies (Mao et al., 2018).

Although there is abundant data showing the geographical distribution, host range, and infection sites of this pathogen, the mechanisms that lead to the establishment and progression of *H. ovis* infections remain unexplored. As shown in an invertebrate infection model, *H. ovis* strains originating from the uterus of dairy cows can display varying degrees of virulence (Cunha et al., 2023). Based on the virulence phenotypes from that study, whole genome comparative analyses identified potential high virulence determinants in this organism, including Zinc ABC transporters, two hypothetical proteins, and a pathogenicity island (Cunha et al., 2023). These comparative analyses created a blueprint for investigating *H. ovis* pathogenic capabilities, but the role these virulence factors may play in disease pathogenesis remains unexplored.

One of the most prevalent and costly animal diseases associated with *H. ovis* is metritis in dairy cows (Cunha et al., 2018; Pérez-Báez et al., 2021). This disease is characterized by acute uncontrolled opportunistic bacterial proliferation within the uterus in the face of immune dysregulation, leading to painful inflammation, impaired fertility, and sometimes death. The primary causative agents of metritis have not been clearly identified, but studies have shown that *Bacteroides pyogenes*, *Fusobacterium necrophorum*, *Porphyromonas levii*, and *Helcococcus ovis* are of importance in its etiology (Cunha et al., 2018; Jeon et al., 2015; Jeon and Galvão, 2018). Among these organisms, *H. ovis* is atypical in that it is a Gram-positive facultative anaerobic bacterium within an infection environment dominated by Gram-negative obligate anaerobes. Exploring the genomic features and diversity of this bacterium is a key step in unraveling its role in the pathogenesis of mixed infections.

Pangenomic analyses can provide valuable insights into the genomic diversity, virulence factors, and potential antimicrobial resistance profiles of bacterial populations. By examining the pangenome, which includes the core genome shared by all isolates and the accessory genome comprising genes unique to specific isolates, we can identify genetic variations that may be associated with virulence or adaptations to the uterine microenvironment in health or disease. A pangenomic analysis of *H. ovis* isolates from the uterus of dairy cows can offer a comprehensive view of the bacterium’s genomic characteristics and shed light on its pathogenic potential, host adaptation, and implications for antimicrobial treatment options.

In this study, we sequenced the genomes of 35 *Helcococcus* clinical isolates from the uterus of dairy cows and tested their antimicrobial resistance and biochemical phenotypes. Phylogenetic and average nucleotide identity analyses placed 4 *Helcococcus* isolates within a cryptic clade, representing an undescribed species. We applied whole genome comparative analyses to explore the pangenome, resistome, virulome, and taxonomic diversity of the remaining 31 *H. ovis* isolates.

## Results

### *Helcococcus ovis* isolation is associated with metritis

Of the 38 cows evaluated for metritis diagnosis, 21 were healthy and 17 were diagnosed with metritis. As shown in Table 1, six healthy cows and 15 cows with metritis were culture-positive for *H. ovis*. We used Fisher’s exact test to examine the relationship between metritis and the presence of live *H. ovis* in the uterus, which showed a statistically significant association (*p* < 0.001). The odds ratio (OR) for cows with metritis was found to be 18.75 (95% confidence interval: 3.14–92.85), indicating an 18-fold increased risk of being culture-positive for *H. ovis* compared to healthy cows. Clinical data for the cows used for this study and for those associated with strains included from previous studies is presented in Supplementary File S1.

**Table 1:** Two-by-two contingency table of *H. ovis* isolation in healthy cows and cows with metritis.

	Healthy	Metritis	Row Sum
*H. ovis* positive	6	15	21
*H. ovis* negative	15	2	17
Column sum	21	17	

*p* < 0.001, OR = 18.75 (95% CI: 3.14–92.85).

### Read quality and assemblies

**Figure 1:**
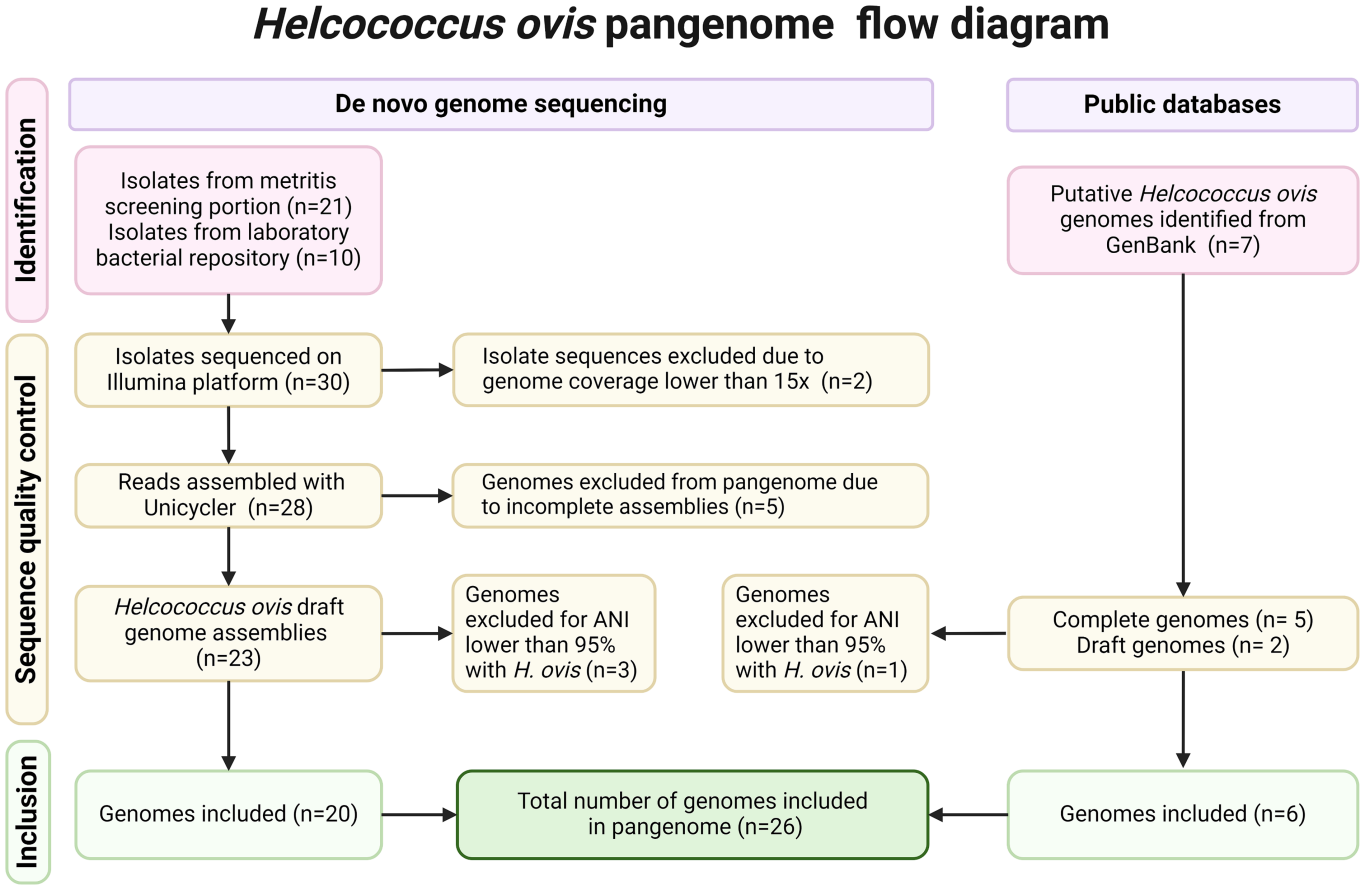
PRISMA flow diagram detailing inclusion and exclusion criteria for each *Helcococcus* strain.

A total of 38 *Helcococcus* sp. genomes were considered for inclusion in the *H. ovis* pangenome analysis. Thirty-one *H. ovis* isolates were selected for Illumina sequencing. These included 21 isolates recovered from the screening portion of this study and 10 strains isolated from the uterus of dairy cows that were not part of the screening portion of the study. Two isolates originating from the screening portion of this study (KG111 and KG115) were excluded from further analysis because the resulting reads did not reach the desired coverage threshold of at least 15x. The remaining 28 sets of Illumina reads had a mean of 163 mega base pairs (Mbp), ranging from 30 to 567 and amean coverage of 90x, ranging from 16x to 308x. Detailed read quality metrics and SRA accession numbers are listed in Supplementary File S2. A flow diagram detailing the provenance and exclusion of all isolates considered in the study is shown in Figure 1.

Unicycler was used to assemble the Illumina reads of the 28 remaining isolates. Of the resulting de-novo assemblies, five (KG101, KG116, KG93, KG118, KG97) resulted in unexpectedly small genome sizes ranging from 1.02 to 1.48 Mbp compared to the expected range of 1.7–1.85 Mbp. These genome assemblies also have fewer than the expected >1,600 coding sequences (CDS) (1234–1,537) and fewer than the expected 33 tRNAs (Brynildsrud et al., 2016; CLSI, 2024; Jain et al., 2018; Martinez et al., 2014; Jeon et al., 2016; Cunha et al., 2019; Gomez et al., 2019) found in *H. ovis*. Therefore, they were excluded from the pangenome analysis. However, they were retained for other analyses since they can provide valuable taxonomic and gene presence information. Draft genomes from two additional *H. ovis* strains from cows with metritis (KG39 and KG40) were retrieved from GenBank. Finally, we also included five complete *H. ovis* genomes (KG36, KG37, KG38, KG104, and KG106) from a previous study (Cunha et al., 2023). Genome assembly statistics for all 35 assembled genomes included in this study and their accession numbers are listed in Supplementary File S3.

### *Helcococcus* cryptic strains

In a recent study we identified a single putative *H. ovis* strain (KG38) whose average nucleotide identity (ANI) with other *H. ovis* strains is lower than the suggested 96% threshold for same species determination (Cunha et al., 2023). Although the initial identification of *H. ovis* isolates for this experiment was conducted based on 16S rRNA sequence identity comparisons, 16S rRNA sequence variations are often not specific enough to discriminate between closely related species (Williamson et al., 2022). To screen for the presence of any cryptic strains among our assembled genomes, we created a maximum likelihood phylogenetic tree and evaluated all-*vs*-all ANI relationships between all the strains in this study.

### Taxonomy, ANI and DDH

**Figure 2:**
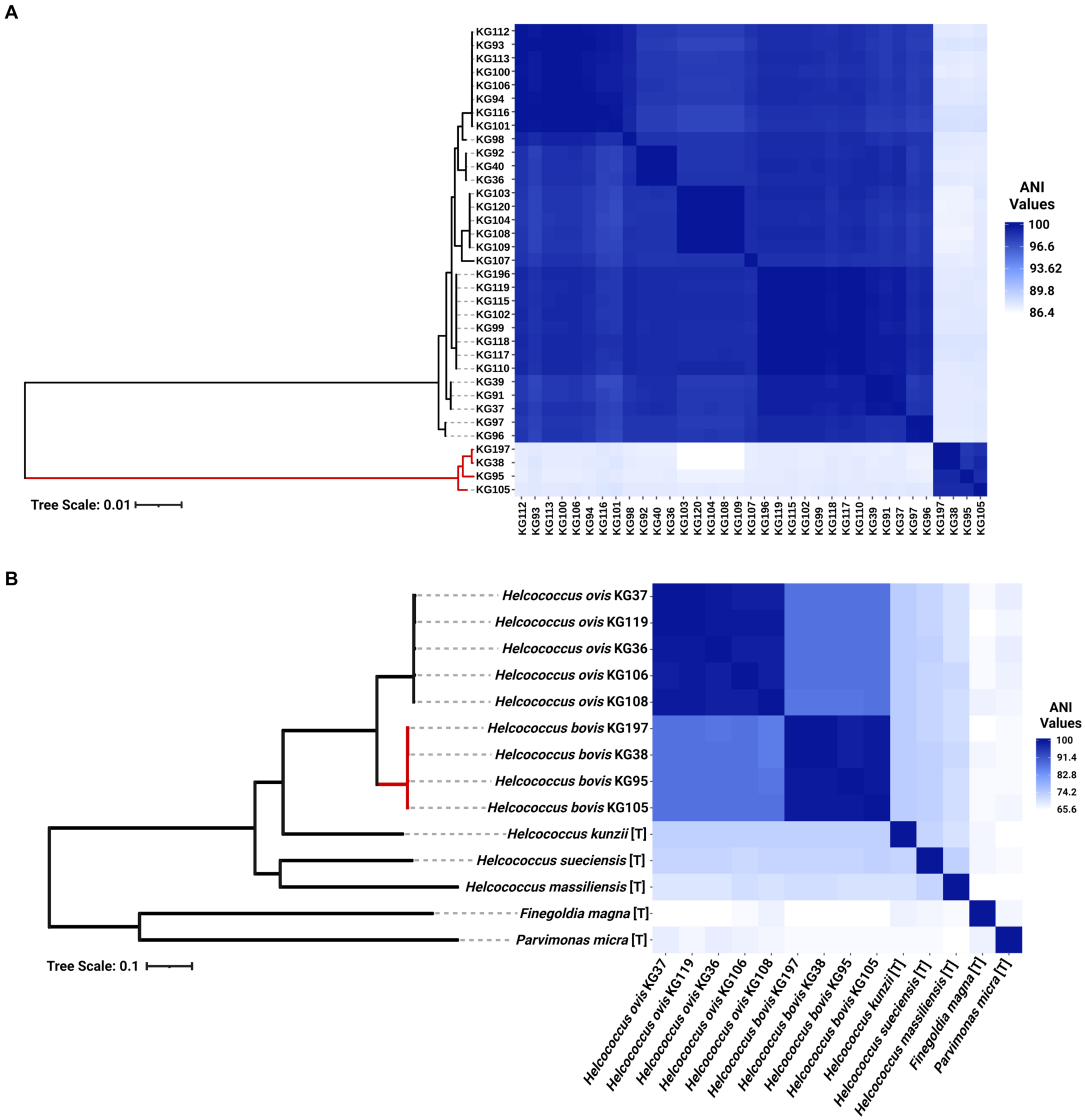
**(A)** Heat map of whole genome average nucleotide identity based on BLAST+ (ANIb) and maximum-likelihood phylogenetic tree of 35 *Helcococcus* strains included in this study. The clade colored in red represents cryptic *Helcococcus* clade. **(B)** Heat map of whole genome average nucleotide identity based on BLAST+ (ANIb) and maximum-likelihood phylogenetic tree of the selected genomes of *Helcococcus ovis* and *Helcococcus bovis*, the type strains of the remaining species of the *Helcococcus* genus, and the type species of the most closely related genera to *Helcococcus*, *Finegoldia* and *Parvimonas*. [T] denotes the genome of a type organism.

As shown in Figure 2A, four (KG38, KG95, KG105, and KG197) of the 37 strains included in this study cluster together in a clade forming an outgroup from the remaining 33 *H. ovis* strains. These strains have ANIs lower than 90% with the rest of the *H. ovis* species and also a higher than 96% ANI between each other. Although these four cryptic strains are closely related to *H. ovis,* their taxonomic position within the genus *Helcococcus* and family *Peptoniphilaceae* is unclear. We selected one representative *H. ovis* strain for each subclade within the species taxon and created a maximum likelihood phylogenetic tree which also includes the type strains for all species of the *Helcococcus* genus as well as the type species for the most closely related genera to *Helcococcus*: *Finegoldia* and *Parvimonas*. Figure 2B shows this phylogenetic tree alongside a heat map of ANI values between *H. ovis* strains and type strains of other close species and genera. Strains KG38, KG95, KG105, and KG197 form a cryptic clade within *Helcococcus*. Having less than 95% ANI with every other species of the *Helcococcus* genus is evidence that these strains represent a distinct novel species. Furthermore, having a higher than 70% ANI with the type strains of other *Helcococcus* species and less than 70% ANI with *Finegoldia magna* and *Parvimonas micra* is robust evidence that these strains belong to the genus *Helcococcus*. These observations are also supported by the maximum likelihood phylogenetic tree that was constructed with 232 orthologous genes shared across stains. Finally, to further support the ANI analyses we evaluated the genomic relatedness between strains for which we have complete genomes with in-silico DNA–DNA hybridization (DDH) using the standard 70% DDH species threshold. We compared *Helcococcus* strains KG36, KG37, KG104, KG106, and KG38. The results, presented in Supplementary File S4, support the ANI results showing KG38 does not belong to the *Helcococcus ovis* species.

**Figure 3:**
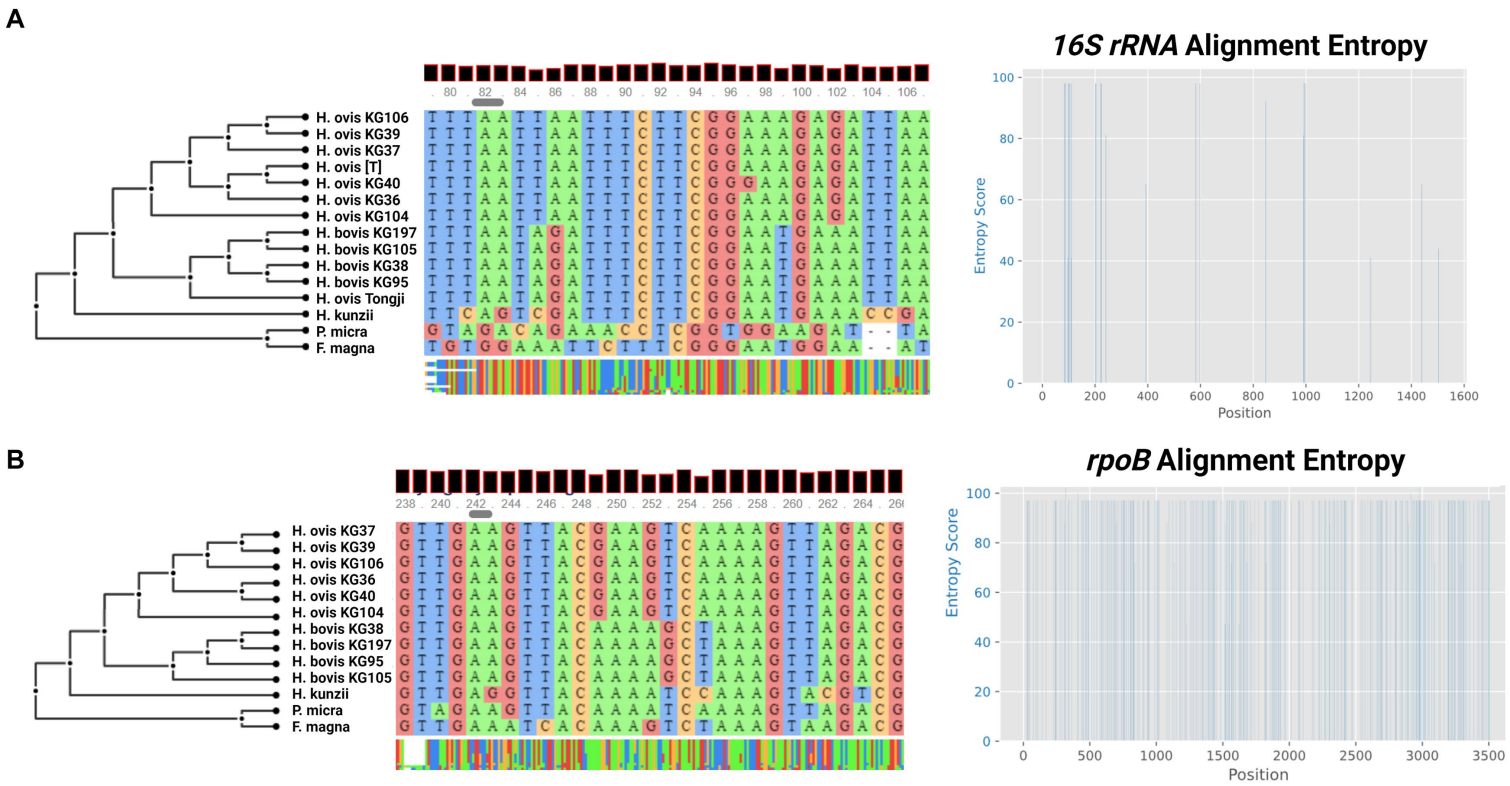
**(A)** Multiple sequence alignment and sequence entropy plot of near-complete *Helcococcus ovis* 16S rRNA from this study, the *Helcococcus ovis* Tongji strain, and the *Helcococcus ovis* type strain. **(B)** Multiple sequence alignment and sequence entropy plot of *rpoB* sequences from this study. Alignment windows display examples of areas of sequence entropy that can be used to differentiate between *Helcococcus ovis* and *Helcococcus bovis*.

Although the only publicly available whole genome sequences of *H. ovis* are from isolates associated with metritis in Holstein dairy cows, there are few publicly available near-complete *H. ovis* 16S rRNA sequences. As shown in Figure 3A, a multiple sequence alignment of near-complete *H. ovis* 16S rRNA sequences from this study, the Tongji strain, and the *H. ovis* type strain is able to discriminate between the core *H. ovis* clade and the cryptic strains. However, as shown in Figure 3A, these differences are driven by single nucleotide polymorphisms in hypervariable regions V2 and, to a lesser extent, V6. Based on this multiple sequence alignment, the *H. ovis* Tongji strain can also be considered a member of the cryptic *Helcococcus* sp. clade.

In an attempt to identify a single marker gene to resolve the two *Helcococcus* sp. groups we also extracted the *rpoB* gene from the same genome assemblies in this study. Although there are no publicly available *rpoB* sequences for *H. ovis*, it is an often-used core gene for bacterial phylogenetic analyses. As shown in Figure 3B, the multiple sequence alignment is able to discriminate between the core *H. ovis* clade and the cryptic strains while also having areas of sequence entropy across the gene, making it a better candidate single marker gene than 16S rRNA for Helcococcus species differentiation.

### Proteome comparison

**Figure 4:**
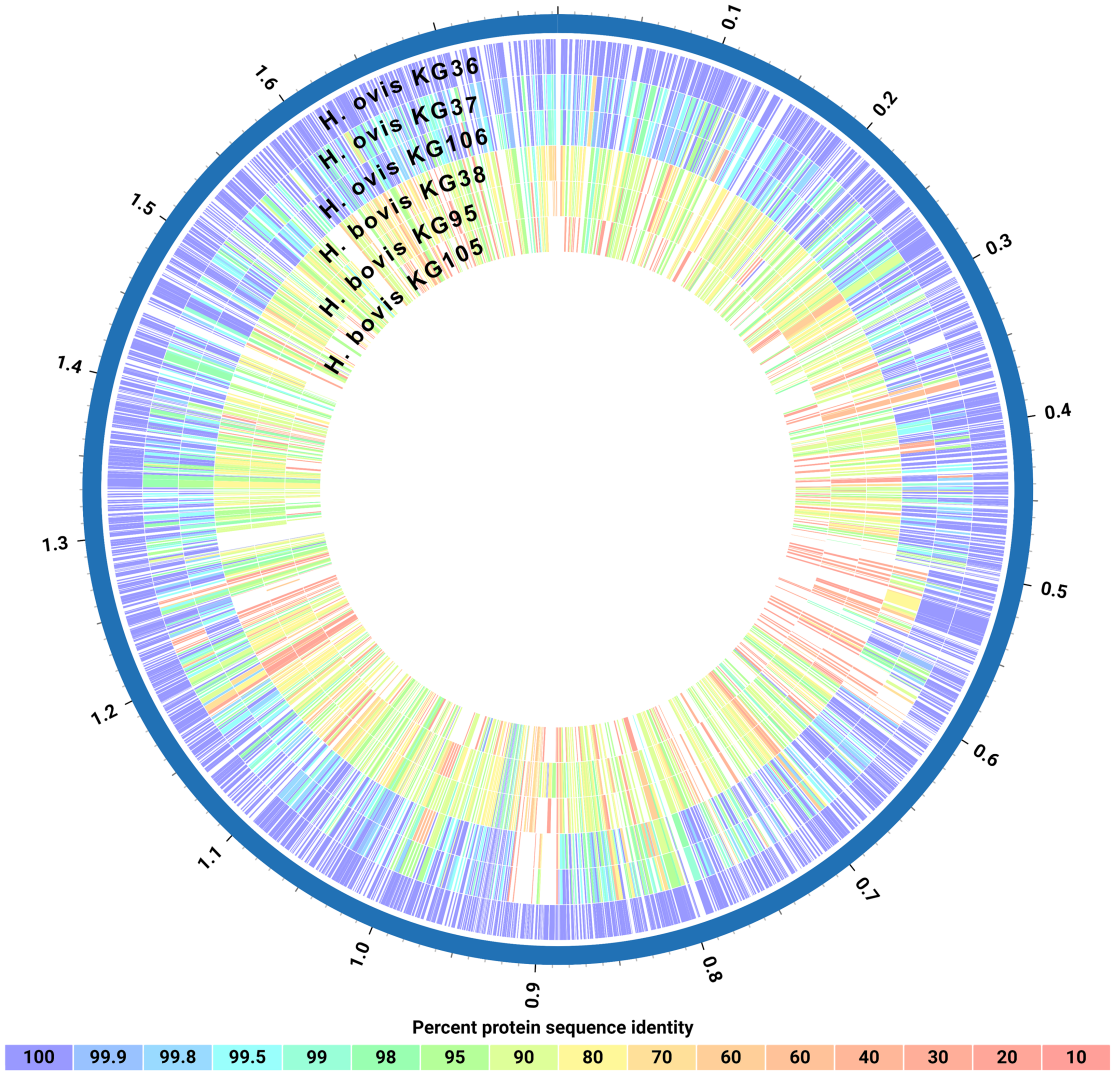
Circos plot of protein sequence alignments of three *Helcococcus ovis* and three *Helcococcus bovis* strains. Percent protein sequence identities were calculated against the proteome of reference strain *Helcococcus ovis* KG36.

Finally, as shown in Figure 4, a protein blast alignment between the complete proteomes of three representative *H. ovis* strains (KG36, KG37, and KG106) and three of the cryptic strains (KG38, KG95, and KG105) illustrates that the cryptic strains have protein sequence identities with the reference *H. ovis* KG36 as low as 80–70% across their genomes.

### Phenotypic characteristics

**Figure 5:**
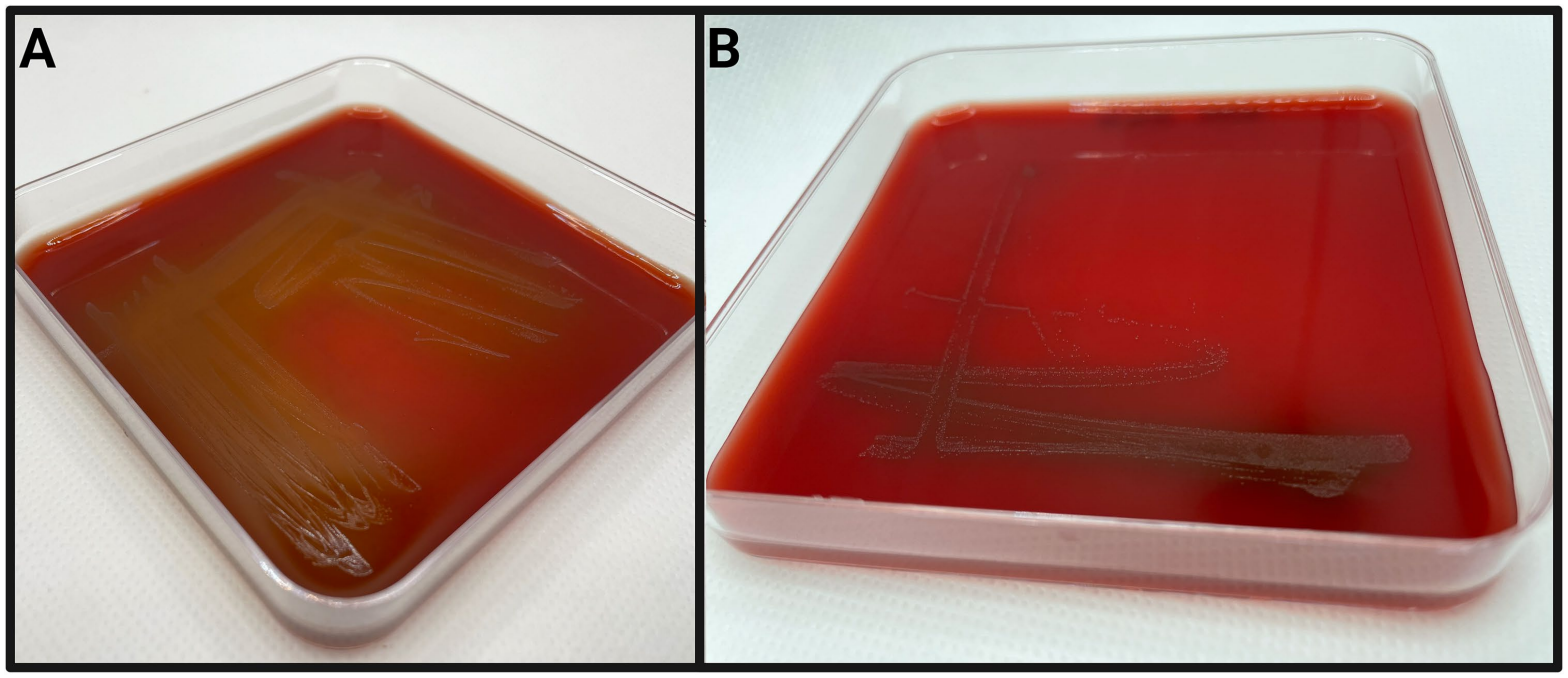
Examples of *Helcococcus ovis* KG37 **(A)** and *Helcococcus bovis* KG38 **(B)** culture on tryptone soy agar with 5% defibrinated sheep’s blood and 0.001% pyridoxal HCl after 72 h of incubation.

Phenotypically, the cryptic *Helcococcus* sp. strains are Gram-positive facultative anaerobic cocci that depend on pyridoxine supplementation for growth *in vitro*. They can be cultivated at 36–38°C on tryptone soy agar (TSA) with 5% defibrinated sheep blood and 0.001% pyridoxal HCl. After 72–96 h of incubation, they form pinpoint transparent colonies morphologically indistinguishable from *H. ovis* and displaying little to no alpha hemolysis. As shown in Figure 5, cryptic strain KG38 displays weak hemolysis on blood agar when compared to *H. ovis* strains. In liquid medium, both *H. ovis* and the cryptic strains grow well in brain heart infusion (BHI) broth supplemented with 0.1% Tween80 and 0.001% pyridoxal HCl.

As shown in Table 2, eight isolates were assessed to identify their enzymatic activity. *H. ovis* strains KG36, KG37, KG104, and KG106, and cryptic strains KG38, KG95, 105, and KG197 exhibited positive results for both alanine arylamidase and L-proline arylamidase. However, in contrast to the cryptic strains, *H. ovis* strains also demonstrated positive results for at least one of the following: tyrosine arylamidase (3/4), Beta galactopyranosidase (2/4), D-mannose (3/4), or D-maltose (1/4). Cryptic strain KG38 was the sole strain positive for leucine arylamidase and alanyl-phenylalanyl-proline arylamidase. Although the Vitek 2 Gram-Positive ID card is capable of identifying *Helcococcus kunzii* based on its biochemical profile, it is not designed to identify *H. ovis*. As a result, all but one tested strain produced “low confidence” or “unknown” species identification. Cryptic strain KG38 was misidentified as 99% probability “*Dermacoccus nishinomiyaensis*/*Kytococcus sedentarius*.” These results indicate that differentiating between *H. ovis* and the cryptic *Helcococcus* strains may be possible based on the absence of specific enzymatic activity beyond alanine arylamidase and L-proline arylamidase. However, a larger sample size is needed to draw any conclusions regarding the differential enzymatic activities of these two clades.

**Table 2:** Biochemical characteristics and Vitek2 identification results of *Helcococcus* sp. isolates.

Isolate	APPA	LeuA	AlaA	ProA	TyrA	BGAR	dMNE	dMAL	Vitek2 ID
*Helcococcus ovis* KG104	−	−	**+**	**+**	**+**	**+**	−	−	Low Discrimination
*Helcococcus ovis* KG106	−	−	**+**	**+**	**+**	−	**+**	−	Low Discrimination
*Helcococcus ovis* KG36	−	−	**+**	**+**	−	**+**	**+**	−	Unidentified
*Helcococcus ovis* KG37	−	−	**+**	**+**	**+**	−	**+**	**+**	*Granulicatella elegans* (95% Probability)
*Helcococcus bovis* KG38	**+**	**+**	**+**	**+**	−	−	−	−	*Dermacoccus nishinomiyaensis*/ *Kytococcus sedentarius* (99% Probability)
*Helcococcus bovis* KG95	−	−	**+**	**+**	−	−	−	−	Low Discrimination
*Helcococcus bovis* KG105	−	−	**+**	**+**	−	−	−	−	Low Discrimination
*Helcococcus bovis* KG197	−	−	**+**	**+**	−	−	−	−	Low Discrimination

Only tests with at least one positive result are included. A complete list of biochemical tests is presented in Supplementary File S5.

According to these phenotypic results, alanine and proline hydrolysis are a core enzymatic activity of both *H. ovis* and *H. bovis.* Accordingly, all 8 strains carry Xaa-Pro Aminopeptidase (EC 3.4.11.9) genes that can specifically hydrolyze proline-containing peptides. All these strains also contain genes encoding Aminopeptidase C (EC 3.4.22.40) which can hydrolyze alanine. There are no genes present only in *H. bovis* K38 that could explain its unique Ala-Phe-Pro and Leucine hydrolysis capacity. However, this strain carries Aminopeptidase S (Leu, Val, Phe, Tyr preference) (EC 3.4.11.24) and Xaa-Pro Aminopeptidase (EC 3.4.11.9) which, in specific contexts, can hydrolyse leucine and Ala-Phe-Pro, respectively. (Rawlings et al., 2010). All 8 strains have genes associated with mannose utilization (mannose-specific components of the PTS system (EC 2.7.1.191)) and maltose utilization (4-alpha-glucanotransferase (amylomaltase) (EC 2.4.1.25)). *H. ovis* KG36 is the only strain that can utilize mannose and also carries a second copy of the gene encoding the mannose-specific components of the PTS system (EC 2.7.1.191). There is significant overlap of genes potentially associated with specific phenotypic tests in these bacteria. Gene expression experiments in the presence of the tested substrates with a wider number of isolates may be necessary to more accurately identify the genes associated with specific phenotypes.

### *H. ovis* Pangenome construction and associations

**Figure 6:**
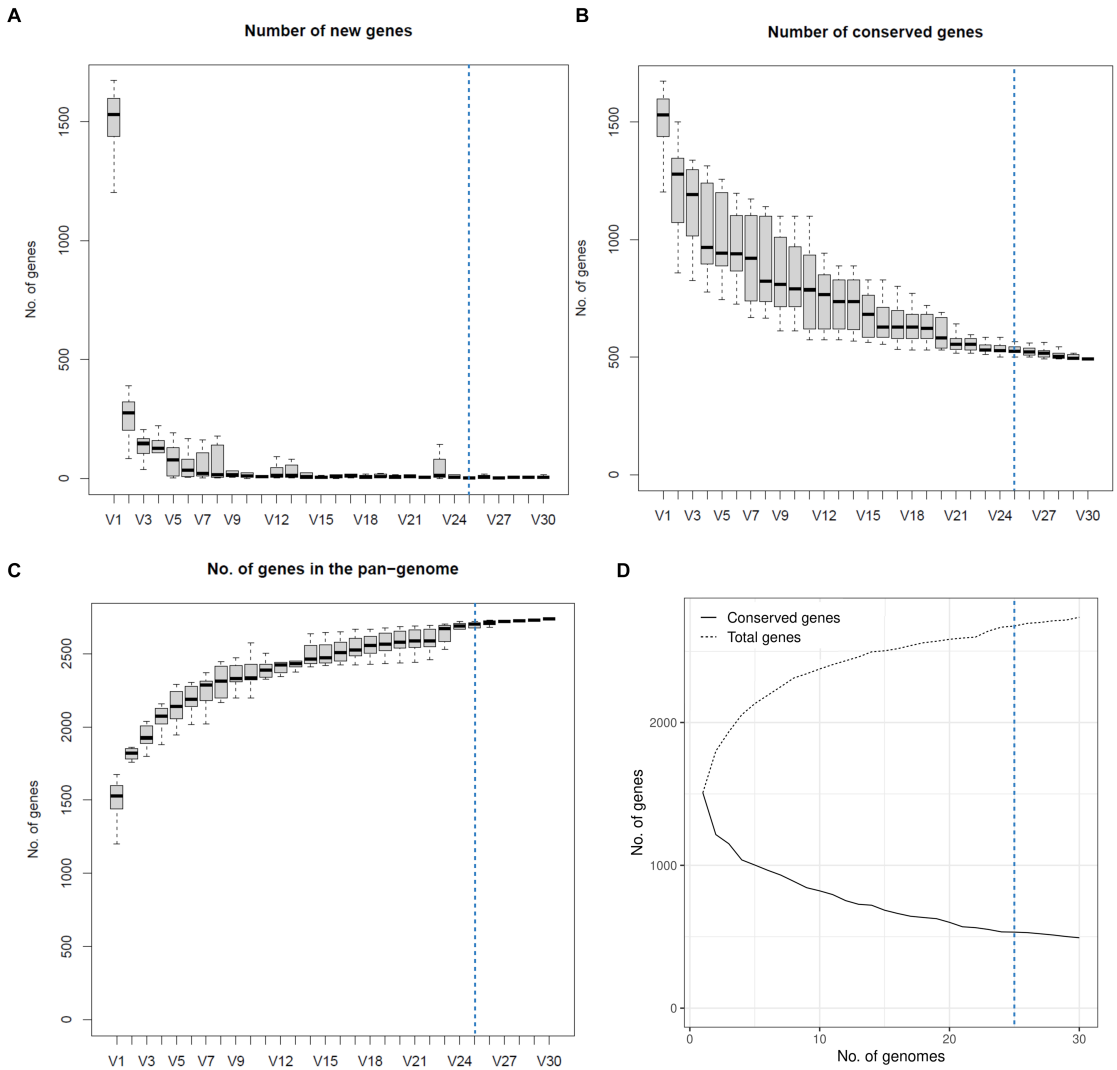
Plots depicting how the pangenome varies as genomes are randomly added to the pangenome construction. The dashed blue line marks the 25-genome threshold selected for this study. **(A)** Number of new genes as genomes are added to the pangenome. **(B)** Number of conserved genes as genomes are added to the pangenome. **(C)** Total number of genes as genomes are added to the pangenome. **(D)** Number of conserved and total genes as genomes are added to the pangenome.

**Figure 7:**
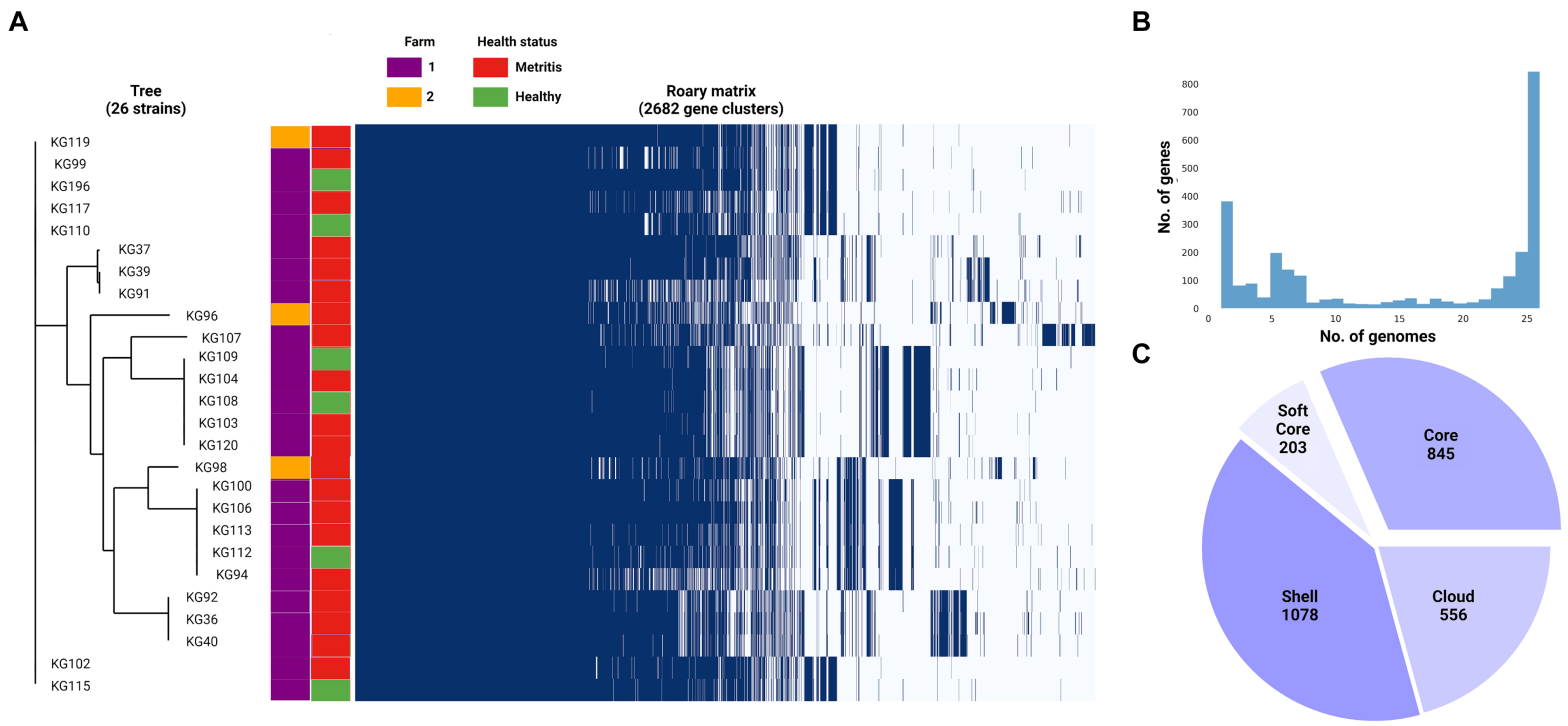
**(A)** *Helcococcus ovis* pangenome gene matrix depicting the 2,682 gene clusters identified by Roary. **(B)** Pangenome frequency plot depicting how many gene clusters are found in only 1 to only 25 genomes. **(C)** Pie chart summarizing the pangenome structure.

The four genomes belonging to the cryptic strains were excluded from pangenomic analyses as this study aims to explore the pangenome of *H. ovis*, the *Helcococcus* species associated with metritis in dairy cows. A total of 31 genomes were initially included in the pangenome construction. However, there were low-quality assemblies that did not result in complete enough genomes to warrant inclusion into the pangenome. As shown in Figure 6, the number of new genes in the pan-genome plateaus after 25 genomes are included. We therefore excluded the five lowest-quality genome assemblies from the pangenome construction and retained 26 assemblies in the analysis. The exclusion of these low-quality assemblies resulted in the core genome expanding from 683 to 1,045 gene families. The resulting core, soft core, shell, and cloud genomes are shown in Figure 7. In short, the *H. ovis* pangenome consists of 845 core genes (99% ≤ strains ≤ 100%), 203 soft core genes (95% ≤ strains <99%), 1,078 shell genes (15% ≤ strains <95%), and 556 cloud genes (0% ≤ strains <15%). The final *H. ovis* pangenome includes 20 strains from cows with metritis and six strains from healthy cows. The complete *H. ovis* pangenome, including the gene presence and absence table, is presented in Supplementary File S6.

Based on the significant differences in the bacterial community composition and uterine metabolome in uteri of healthy dairy cows and those with metritis, we were interested in finding genes that are enriched in the metritis or healthy groups of *H. ovis* strains (Casaro et al., 2024). We ran a Scoary (Brynildsrud et al., 2016) analysis of the 26-strain *H. ovis* pangenome using metritis as the trait of interest. As presented in Supplementary File S7, the initial analysis using a naïve *p*-value identified 3 hypothetical proteins and 3 annotated proteins that were overrepresented in the metritis group. However, due to the large number of pairwise comparisons, applying Bonferroni or Benjamini-Hochberg corrections resulted in *p*-values of 1, nullifying the statistical significance of these findings. Using a significance level of *p* < 0.05 we did not find any gene group overrepresented in either of the host disease groups.

### Virulome

**Figure 8:**
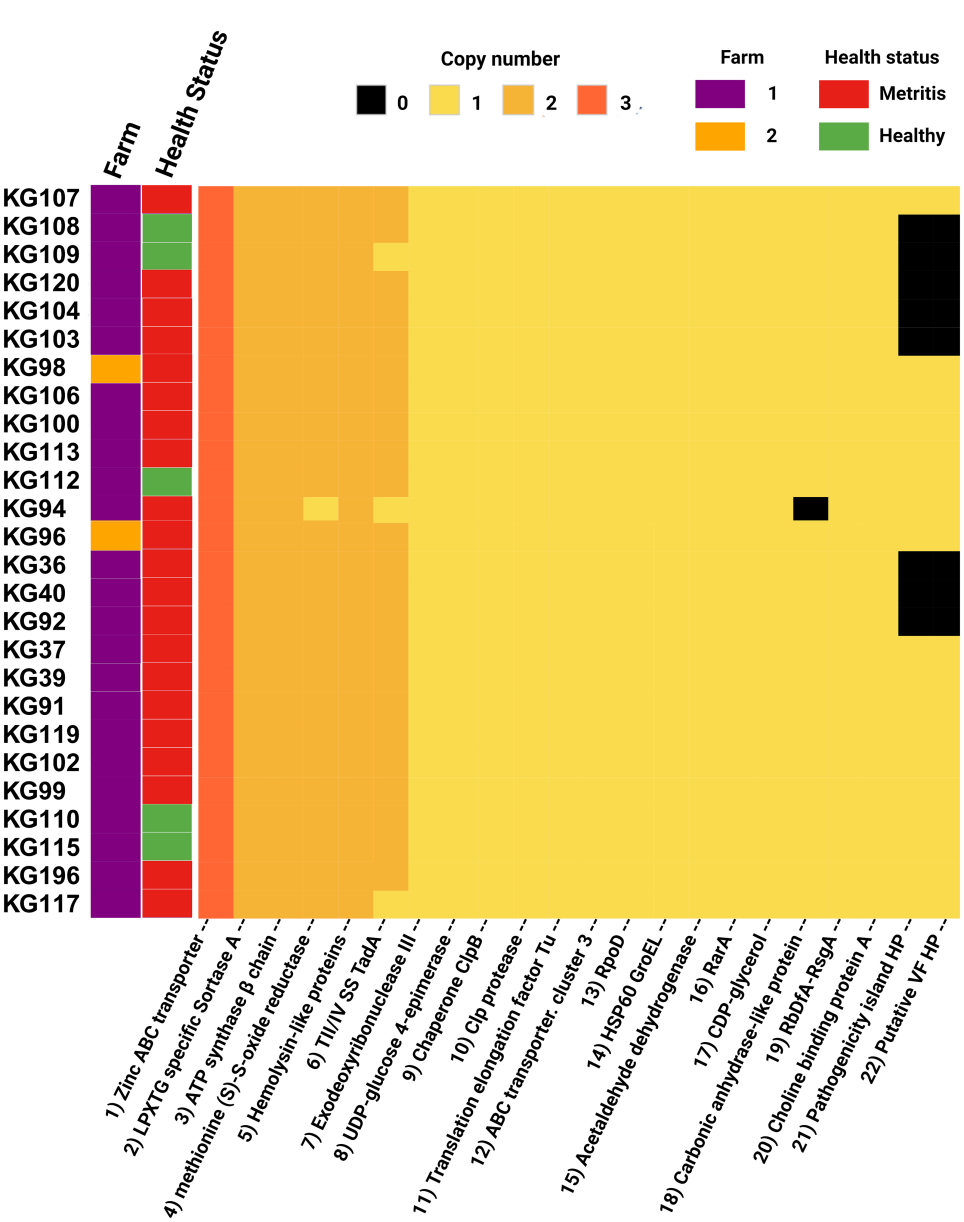
Heat map of virulence factors of *Helcococcus ovis* strains explored in this study.

After using Abricate for mass screening of virulence factors in the 26 *H. ovis* genomes against the Virulence Factor Database, no positive hits were returned. To further investigate the virulome of 26 *H. ovis* strains in this study, we curated a set of 22 putative virulence factor genes based on previous comparative genome analyses (Cunha et al., 2023). The resulting virulome is presented in Figure 8 as a heat map. There is no observable pattern in the presence or absence of virulence factors in these strains in relation to the health status of the host or farm location.

Two hypothetical proteins and a pathogenicity island have been recognized as potential high virulence determinants of *H. ovis* in invertebrate infection models (Cunha et al., 2023). These high virulence determinant CDS are found in 69% (Jeon et al., 2015) of the strains in this study and are absent in only eight strains. Both the hypothetical proteins and the pathogenicity island are co-occurring in every genome where they are present and are altogether absent in the remaining strains. We used mauve to visually inspect the spatial distribution of these co-occurring high virulence determinants in the two complete *H. ovis* genomes where they are present. In both KG37 and KG106, the two hypothetical protein CDS are found closely associated with a ZnuABC locus located more than 500,000 base pairs away from the co-occurring pathogenicity island. To further explore the cause of the co-occurrence and co-absence of these virulence determinant CDS we identified and excluded loci containing elevated densities of base substitutions in the 26 genomes and built an approximately-maximum-likelihood phylogenetic tree (Supplementary File S8). The clades that do not contain the high virulence determinant CDS seem to be spread across the tree, showing that the co-occurring CDS are not restricted to a single lineage but are found in multiple, more distantly related lineages.

### Resistome and plasmids

**Figure 9:**
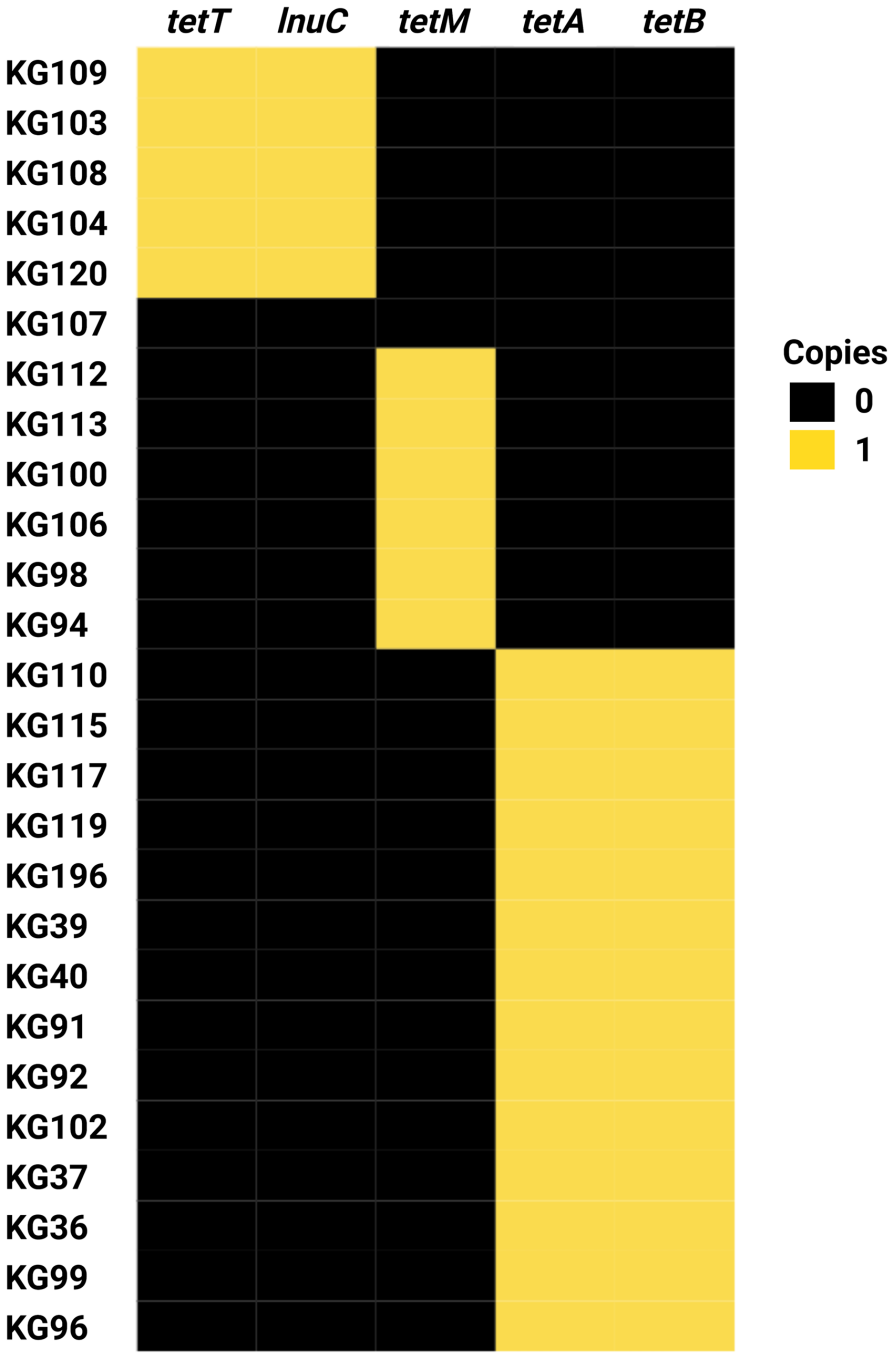
Heat map of antimicrobial resistance gene profiles of *Helcococcus ovis* strains sequenced in this study.

We also used Abricate for mass screening of acquired antimicrobial resistance genes (ARGs) against the Comprehensive Antibiotic Resistance Database. The search was limited to acquired resistance genes because not enough experimental data is available for *H. ovis* to evaluate resistance-associated point mutations. We also screened for known plasmid sequences by querying against the PlasmidFinder database. The results of these analyses are presented in Figure 9.

Strain KG107 is the only one of the 30 screened genomes that does not contain any acquired ARGs. Nine *H. ovis* strains carry only *tetM*, 15 strains carry both *tetA* and *tetB*, and five strains carry both *tetT* and *lnuc*. With the exception of *lnuc,* which confers resistance to lincosamides, all other ARGs found in this experiment confer resistance to tetracyclines. Acquired antimicrobial resistance genes *tetA* and *tetB* are, in all strains, located within a prophage region commonly found within *H. ovis* genomes. Similarly, *tetT* and *lnuC* are found in conjunction within a prophage region in all strains where they occur. This suggests prophage integration events are a significant driver of ARGs acquisition in *H. ovis*. Alternatively, *tetM* is located within a previously described integrated plasmid region (repUS43_1_CDS12738(DOp1)), often found in *Streptococcus* spp.

A total of ten strains were selected to be evaluated for resistance to 22 clinically relevant antimicrobials. Subsets of strains from each AMR genotype including *tetM* only (KG100, KG106, and KG113), *tetA*/*tetB*(KG36, KG37, KG92, KG196), *tetT*/*lnuC* (KG104, KG109, KG120), and none (KG107), were selected for minimum inhibitory concentration (MIC) testing. As shown in Table 3, MICs are reported in μg/mL without antimicrobial resistance breakpoint interpretations because there are currently no interpretive standards established by the Clinical and Laboratory Standards Institute (CLSI) for *H. ovis* in the uterus of cattle. We used the MIC results of strain KG107 as the wild-type reference since it was the only isolate that does not carry any known ARG. Strains carrying any tetracycline resistance gene (*tetT, tetM*, or *tetA*/*tetB*) had a higher MIC for tetracycline. The wild-type strain had a tetracycline MIC ≤0.250 μg/mL, and all other strains had a tetracycline MIC ≥1.0 μg/mL. Although strains carrying *tetM* or *tetT* displayed resistance to doxycycline and minocycline compared to the wild type, strains carrying *tetA*/*tetB* did not follow the same pattern. None of the *tetA*/*tetB* positive strains had increased resistance to minocycline, and their resistance to doxycycline was inconsistent and less than that of *tetM* and *tetT* positive strains. Although MIC for lincomycin were not evaluated, the strains that carry *lnuC* (KG104, KG109, KG120) did not show resistance to clindamycin, the only lincosamide tested.

**Table 3:** Antimicrobial resistance profiles of *Helcococcus* sp.

	*tetA/tetB*				*tetM*			*tetT/lnuC*			None
	KG 37	KG 36	KG 92	KG 196	KG 113	KG 100	KG 106	KG 104	KG 109	KG 120	KG 107
Amikacin	≤16	≤ 16	≤ 16	≤ 16	≤ 16	≤ 16	≤ 16	≤ 16	≤ 16	≤ 16	≤ 16
Amox/Clav	≤0.25	≤0.25	0.5	≤0.25	≤0.25	≤0.25	≤0.25	≤0.25	0.5	0.5	≤0.25
Ampicillin	≤0.25	≤0.25	0.5	≤0.25	≤0.25	≤0.25	≤0.25	≤0.25	0.5	0.5	≤0.25
Cefazolin	≤ 2	≤ 2	≤ 2	≤ 2	≤ 2	≤ 2	≤ 2	≤ 2	≤ 2	≤ 2	≤ 2
Cefovecin	2	2	4	1	1	1	0.5	2	4	4	0.5
Cefpodoxime	≤ 2	≤ 2	≤ 2	≤ 2	≤ 2	≤ 2	≤ 2	≤ 2	≤ 2	≤ 2	≤ 2
Cephalothin	≤ 2	≤ 2	≤ 2	≤ 2	≤ 2	≤ 2	≤ 2	≤ 2	≤ 2	≤ 2	≤ 2
Chloramphenicol	≤ 8	≤ 8	≤ 8	≤ 8	≤ 8	≤ 8	≤ 8	≤ 8	≤ 8	≤ 8	≤ 8
Clindamycin	≤ 0.5	≤ 0.5	≤ 0.5	≤ 0.5	≤ 0.5	≤ 0.5	≤ 0.5	≤ 0.5	≤ 0.5	≤ 0.5	≤ 0.5
Doxycycline	0.25	≤ 0.125	0.5	≤0.125	>0.5	>0.5	>0.5	>0.5	>0.5	>0.5	≤0.125
Enrofloxacin	≤ 0.25	≤ 0.25	≤ 0.25	≤ 0.25	≤ 0.25	≤ 0.25	≤ 0.25	≤ 0.25	≤ 0.25	≤ 0.25	≤ 0.25
Erythromycin	1	1	1	1	2	1	1	0.5	2	1	1
Gentamicin	≤ 4	≤ 4	≤ 4	≤ 4	≤ 4	≤ 4	≤ 4	≤ 4	≤ 4	≤ 4	≤ 4
Imipenem	≤ 1	≤ 1	≤ 1	≤ 1	≤ 1	≤ 1	≤ 1	≤ 1	≤ 1	≤ 1	≤ 1
Marbofloxacin	≤ 1	≤ 1	≤ 1	≤ 1	≤ 1	≤ 1	≤ 1	≤ 1	≤ 1	≤ 1	≤ 1
Minocycline	≤ 0.5	≤ 0.5	≤ 0.5	≤ 0.5	2	2	1	>2	>2	>2	≤ 0.5
Oxacillin	≤0.25	≤0.25	>2	>2	>2	>2	≤0.25	≤0.25	>2	>2	2
Penicillin	0.125	≤0.06	0.25	≤0.06	≤0.06	≤0.06	≤0.06	≤0.06	0.25	0.25	0.125
Rifampicin	≤ 1	≤ 1	≤ 1	≤ 1	≤ 1	≤ 1	≤ 1	≤ 1	≤ 1	≤ 1	≤ 1
Tetracycline	>1	>1	>1	1	1	>1	>1	>1	>1	>1	≤0.25
Trim./Sulfa	≤ 2	≤ 2	≤ 2	≤ 2	≤ 2	≤ 2	≤ 2	≤ 2	≤ 2	≤ 2	≤ 2
Vancomycin	≤ 1	≤ 1	≤ 1	≤ 1	≤ 1	≤ 1	≤ 1	≤ 1	≤ 1	≤ 1	≤ 1

Minimum inhibitory concentration values are in microgram/milliliter (μg/mL). The rows of tetracycline class antibiotics are highlighted in gray.

Finally, as shown in Table 3, strains KG92, KG196, KG113, KG100, KG107, KG109, and KG120 showed resistance oxacillin without carrying any ARG known for conferring resistance to beta-lactams. We ran a Scoary analysis using a resistance threshold of ≥0.5 for oxacillin, using the CLSI soft-tissue cutoffs for non-*Staphylococcus aureus* staphylococci in dogs and cats as an approximation (CLSI, 2024). We used the Benjamini-Hochberg adjusted *p*-values to identify the genes most overrepresented in a specific host group. We did not find any gene group overrepresented in either of the host groups using a significance level of *p* < 0.05. This suggests that beta-lactam resistance in *H. ovis* may be mediated by chromosomal mutation resistance instead of by the presence or absence of antimicrobial resistance genes. We used the BV-BRC comparative systems pathway analysis tool to identify genes associated with peptidoglycan biosynthesis. We then identified 3 penicillin binding proteins in *H. ovis*, including Cell division protein FtsI [Peptidoglycan synthetase] (EC 2.4.1.129), Peptidoglycan D,D-transpeptidase MrdA (EC 3.4.16.4), and Multimodular transpeptidase-transglycosylase (EC 2.4.1.129). Multiple sequence alignments of these penicillin binding proteins showed 6 amino acid substitutions in Ftsl, 4 in MrdA, and 5 in Multimodular transpeptidase-transglycosylase,. Although these substitutions could potentially influence oxacillin binding affinity to PBP conferring resistance, there is no amino acid substitutions exclusive to oxacillin resistant strains. Further studies are necessary to identify the cause of the increased oxacillin resistance in these strains.

## Discussion

In this study, we examined the genomes of *H. ovis* strains obtained from the uteri of both healthy dairy cows and those with metritis. Our analysis focused on exploring the pangenome, resistome, virulome, and taxonomic diversity of these strains. Additionally, we sought bacterial genome-wide associations between *H. ovis* gene clusters and metritis in dairy cows.

While the costs of second-generation short-read whole-genome sequencing (WGS) have significantly decreased in the past decade, third generation long-read sequencing remains a less affordable emerging technology. In our study, we opted for the more commonly used approach of employing low-depth Illumina short-read sequencing to maximize the inclusion of a larger number of strains in our analysis. As is evident in our findings, this approach may lead to the loss of genomes due to low coverage and incomplete assemblies. However, this tradeoff is acceptable when considering the low-cost, high-throughput generation of excellent-quality reads (Q > 35).

The *Helcococcus* genus is comprised of five well-described species. However, their genetic diversity remains unexplored due to the limited availability of sequenced genomes. Within this small genus there are still unresolved and contradictory taxonomic classifications. For example, the species *H. pyogenes* was described as a new species isolated from a prosthetic joint infection based on biochemical tests and a partial (518 bp) 16S rRNA sequence identity in 2004 (Panackal et al., 2004). A later study proposed another new species, *H. seattlensis*, isolated from a human with urosepsis also based on 1,512 bp 16S rRNA sequence identity, which also shares 99.4% sequence identity with *H. pyogenes* suggesting it is likely the same species (Chow and Clarridge, 2014). Another taxonomic uncertainty within the genus is the classification of the *H. ovis* Tongji strain, isolated from the only recorded *H. ovis* infection in a human (Mao et al., 2018). This strain displayed an atypical biochemical profile for *H. ovis* and a 98.9% 16S rRNA sequence identity with the *H. ovis* type strain, which led researchers to question its place within the species taxon. Phylogenomic analyses have shown that, based on 16S rRNA sequence identity, the Tongji strain belongs to a subclade of the species also populated by *H. ovis* strain KG38 (Cunha et al., 2023). Whole genome-based multi-locus phylogenomic analyses in this study confirmed these findings and identified three further strains (KG95, KG105, and KG197) belonging to the cryptic clade. Average nucleotide identity, proteome identity, and phenotypic analyses provide robust evidence that these strains belong to a distinct novel species, for which we propose the name *H. bovis* sp. nov. (bo’vis. L. gen. n. bovis of the cow). *H. ovis* and *H. bovis* strains share 87–89% average nucleotide identity with each other placing their relationship in the 0.2% of pairs that fall within the 83–95% ANI valley range (Jain et al., 2018). This makes the relationship between the two species a rare candidate for exploring bacterial speciation mechanisms and the role horizontal gene transfer has on the speciation process. These *H. bovis* strains originated from two geographically separate farms in North Central Florida. They were also retrieved from uteri of both cows with metritis (Collins et al., 1993) and healthy cows (Chow and Clarridge, 2014). However, the sample size in this study is too small to draw conclusions about the association between the presence of *H. bovis* and uterine health status. Strain KG38, part of the novel species group, has been shown to have attenuated virulence when compared to other *H. ovis* isolates (Cunha et al., 2023). Since *H. bovis* occupies a similar biochemical niche as the more virulent *H. ovis* strains, its role as a commensal organism of the reproductive tract is an area that warrants further exploration. Although the multiple sequence alignment of the whole 16 S rRNA gene can discriminate between *H. ovis* and *H. bovis*, the responsible sequence variations are in hypervariable regions V2 and V6 which are not often targeted in metagenomic amplicon studies. This means sequences belonging to *H. bovis* will contribute reads to the amplicon sequence variants classified as *H. ovis* in most metagenomic studies amplifying the V3-V4 hypervariable regions. Unlike the 16S rRNA sequence, *rpoB* has more regions of dissimilarity between *H. ovis* and *H. bovis*, making it a much more useful single-marker gene to resolve these two *Helcococcus* species.

We screened a subset of cows from this study to show that isolation of *H. ovis* from the uterus of dairy cows is strongly associated with metritis. Previous studies have shown that *H. ovis* DNA is more abundant in relative and absolute terms in the uterus of dairy cows with metritis than in healthy cows at the time of metritis diagnosis (Cunha et al., 2018; Jeon et al., 2015). Although all healthy cows have been shown to harbor *H. ovis* genomic DNA (gDNA) in the uterus after parturition, this gDNA is not indicative of the presence of viable bacteria, likely because healthy cows are able to mount adequate immune responses that neutralize these organisms (Martinez et al., 2014; Jeon et al., 2016). Previous to this study, isolation of live *H. ovis* from the uterus of dairy cows had been limited to only cows with metritis, and targeted comparative cultivation screenings have not been conducted (Locatelli et al., 2013; Cunha et al., 2019). These results are a robust addition to the current body of evidence showing *H. ovis* is one of the key organisms in the pathogenesis of metritis in dairy cows at the time of diagnosis.

Our inability to find any *H. ovis* genotype association with metritis is likely due to the fact that metritis is characterized by a dysbiosis of the uterine microbiota that is unlikely to be explained by gene groups within a single component bacterial species (Jeon and Galvão, 2018; Gomez et al., 2019). Furthermore, as is the case in microbial communities in gut dysbiosis, it is possible that *H. ovis* is more prevalent in the diseased uterus because the disease condition widens an independent metabolic niche which the bacterium can then fill without having to play a key role in the necessary steps for the development of disease (Watson et al., 2023). The genome-wide association analyses conducted in this study have been successfully used to find the genetic basis for high penetrance phenotypes in bacteria like virulence (Laabei et al., 2014) or antimicrobial resistance (Ellington et al., 2019), but we were not able to establish any phenotype–genotype link with this approach. In this study we also measured simple phenotypes like hemolysis and pyridoxal dependence *in vitro* for all strains but did not find any insightful phenotypic variation between them.

Since *H. ovis* is not a well-known pathogen or a model organism, there is no experimentally verified virulence factor (VF) data. We found that the putative pathogenicity island and the hypothetical VF associated with a Zinc ABC transporter locus were either co-occurring or altogether absent from *H. ovis* strains. Manual screening of the available complete genomes revealed that these putative VFs are not part of the same operon in the species. This raises the question of whether they are functionally linked or if they display this pattern in our samples by chance. As shown in Supplementary File S8, these putative high virulence determinants are present and absent across different subclades of *H. ovis* and do not show evidence of being driven by the founder effect. According to ProteInfer (Sanderson et al., 2023), both the ZincABC transporter-associated VF and the conserved membrane-spanning protein within the pathogenicity island participate in metal ion binding and transport. This suggests that, as has been shown in *Escherichia coli* (Hall, 2021), these *H. ovis* accessory virulence genes co-occur due to having connected functions, and the resulting phylogenetic distributions are the result of convergent gene loss instead of founder effect.

All the ARGs found in our strains are located within mobile genetic elements like plasmids or prophage regions, which makes *H. ovis* a reservoir of mobile ARGs in a food production setting. All but one *H. ovis* strain (KG107) contain ARGs conferring resistance to tetracyclines. This strain is a valuable clinical isolate since it can be used as a wild-type reference strain with no ARGs to benchmark the susceptibility of *H. ovis* to antimicrobials. Unlike *tetA*/*tetB* positive strains, strains carrying genes encoding the cytoplasmic ribosomal protection proteins TetM or TetT, also have elevated resistance to doxycycline and minocycline. Oxytetracycline and ceftiofur are the only two antimicrobials labeled in the United States for the treatment of metritis in lactating dairy cattle. However, the United States Food and Drug Administration has banned the extra-label use of ceftiofur in animals, and may move toward policies like the Netherlands where the use of ceftiofur administration to agricultural animals is restricted (Kuipers et al., 2016). The alternative, intrauterine oxytetracycline infusions, remains a frequent practice both in clinical and research settings in the United States and Europe (Haimerl et al., 2017; Mileva et al., 2022). Furthermore, dairy operations often use oxytetracycline as prophylactics in heifer rearing or as a treatment for calf pneumonia, a type of infection *H. ovis* has been implicated in (Jost and Sickinger, 2021). Although the MICs for oxytetracycline were not assessed, there is no inherent difference between a tetracycline and an oxytetracycline resistance genes (Chopra and Roberts, 2001), and 96.8% of isolates in this study carry at least one tetracycline resistance gene shown to confer tetracycline resistance *in vitro*. These findings indicate that further studies are needed to evaluate the effectiveness of tetracyclines as a treatment for metritis in dairy cattle.

## Conclusion

This study found that the presence of viable *H. ovis* in the uterus of dairy cows is associated with metritis. However, we found no evidence that a specific *H. ovis* genotype or gene cluster is associated with the disease. Virulence factor comparisons showed two putative high virulence determinants are common but have varying prevalence in these strains with a phylogenetic distribution consistent with convergent gene loss. Based on the genetic dissimilarity and phenotypic characteristics, strains KG38, KG95, KG105, and KG197 represent a novel species of the genus *Helcococcus*, for which we propose the name *Helcococcus bovis* sp. Nov. (bo’vis. L. gen. n. bovis of the cow). The type strain for this species is KG38 (Accession number CP121192). The significance of this species in the context of uterine health remains to be explored. The majority (30/31) of *H. ovis* strains in this study carry antimicrobial resistance genes conferring resistance to tetracyclines, which has significant clinical consequences for the treatment of metritis and other *H. ovis-*associated respiratory infections in cattle. The convergence of widespread ARG-mediated tetracycline resistance in these uterine pathogens and initiatives to phase out the use of ceftiofur to treat metritis reveals an immediate need to find alternative treatments and prevention strategies for this important animal disease.

## Materials and methods

### Metritis diagnosis and uterine sample collection

All procedures involving cows were approved by the Institutional Animal Care and Use Committee of the University of Florida; protocol number 201910623. In this study, a total of 43 lactating Holstein Friesian cows were used. Three cows were from North Florida Holsteins and 40 were from the University of Florida’s Dairy Research Unit, both located in north central Florida. Each cow had uterine discharge collected directly from the uterus with a sterile pipette, and evaluated at 4, 6, and 8 days postpartum. 500 μuL of uterine discharge was suspended in 500 μL of BHI broth with 30% glycerol and stored at −80°C.

The uterine discharge was scored on a 5-point scale (Jeon et al., 2016). Score 1 indicates normal lochia, viscous, clear, red, or brown discharge that was not fetid; Score 2 indicates cloudy mucoid discharge with flecks of pus; Score 3 indicates mucopurulent discharge that was not fetid with less than 50% pus; Score 4 indicates mucopurulent discharge that was not fetid with more than 50% pus; and Score 5 indicates fetid red-brownish, watery discharge. Cows with uterine discharge scores of 1–4 were considered healthy, whereas those with a score of 5 were diagnosed with metritis. Nine cows that had a uterine discharge score of 1 to 4 at the time of sampling but developed metritis sometime in the 21 days after parturition were excluded from microbiological testing.

### Bacteria isolation and identification

To selectively culture *H. ovis* from uterine discharge samples, 20 μL of the discharge suspension was streaked onto *Helcococcus* selective agar. The agar plates were incubated for 72 h at 36°C under aerobic conditions with 6% CO_2_ (Kutzer et al., 2009). Following incubation, individual pinpoint nonpigmented colonies were selected and sub-cultured on tryptone soy agar with 5% defibrinated sheep blood and 0.001% pyridoxal HCl for propagation. Species determination of the isolates was performed via comparative analysis of Sanger sequencing of their 16S rRNA genes.

### Whole genome sequencing

Genomic DNA (gDNA) was extracted using the DNeasy blood and tissue kit following the manufacturer’s instructions (Qiagen). Genomic DNA purity was measured using a NanoDrop 2000 spectrophotometer; final DNA concentration was confirmed with a Qubit 2.0 Fluorometer. DNA integrity was visualized via agarose gel electrophoresis. Library preparation was done with the Nextera XT kit (Illumina, Inc.), following the manufacturer’s instructions, and it was loaded into the MiSeq reagent kit V2. Sequencing was performed on a MiSeq platform (Illumina, Inc.) with a 2 × 250-bp 500-cycle cartridge. Seven previously sequenced strains were also included in this study. Two of them consist of Illumina sequenced draft genomes KG39 (Accession number SRX5460741) and KG40 (Accession number SRX5460742). The remaining five are complete genomes that were hybrid assembled using ONT and Illumina sequencing for genomic comparisons performed in a previous study (Cunha et al., 2023) (KG36, KG36, KG38, KG104, KG106).

### Genome assembly and annotation

After performing quality control with fastp (Chen et al., 2018) the resulting reads were evaluated using MultiQC (v1.14). The minimum coverage threshold for inclusion in the study was set at 15x (Bogaerts et al., 2021). De-novo genome assembly was performed using Unicycler (v0.5.0) (Wick et al., 2017). Assembly quality was assessed using Benchmarking Universal Single-Copy Orthologs (v4.1.2) (Manni et al., 2021). Genome annotations were conducted using Prokka and the genome annotation service in BV-BRC using the RAST tool kit (Seemann, 2014; Brettin et al., 2015).

### Taxonomic analyses

Whole genomes of *Helcococcus* spp. and the type strains of the recognized species within the genera *Helcococcus, Finegoldia, and Parvimonas* were used to create a phylogenetic tree with the BV-BRC codon tree pipeline using 500 single-copy PGFams (Olson et al., 2023). In order to verify that the constructed phylogenetic tree was not affected by recombination events, we used Snippy (v4.6.0) to align Illumina reads of the 26 *H. ovis* genomes using the *H. ovis* KG37 complete genome assembly as reference (Seemann, 2015). We then used Gubbins (v3.3.3) to identify loci affected by recombination and construct a phylogeny based on point mutations outside of these regions (Croucher et al., 2015). Phylogenetic trees were visualized and annotated using Interactive Tree of Life (iTOL v5) webtool (Letunic and Bork, 2021). Average nucleotide identities (ANI) were calculated via BLAST pair-wise comparisons of all sequences shared between two strains (ANIb) using the JSpecies web server (Richter et al., 2016). In-silico DDH analyses were performed between strains where their complete genome was available using GGDC 3.0 using a DDH threshold of 70% for species delineation (Meier-Kolthoff et al., 2022). 16S rRNA gene sequences were extracted from the raw paired-end Illumina reads using phyloFlash (v3.4.2) and *rpoB* genes were extracted from the trycycler-assembled contigs using the BV-BRC Comparative Systems Service (Olson et al., 2023). Extracted nucleotide sequences were aligned using Mafft (v7) and visualized on the BV-BRC Multiple Sequence Alignment and SNP / Variation Analysis Service (Olson et al., 2023; Katoh, 2002).

### Phenotype testing of select isolates

The biochemical profile and antimicrobial susceptibility phenotype of a subset of isolates was assessed at the University of Georgia College of Veterinary Medicine Athens Veterinary Diagnostics Laboratory. using the Vitek2 Gram-positive bacteria ID card for biochemical tests and the Sensititre COMPGP1F plates (ThermoFisher) for MIC testing, according to the manufacturers’ instructions.

For MICs, we inoculated sterile H_2_O with *H. ovis* to achieve a 0.5 McFarland; 10 uL of the inoculum was added to 10 mL of Mueller-Hinton broth containing lysed horse blood and supplemented with 0.1 mg of pyridoxal HCL. Finally, 50 uL of the Mueller-Hinton broth containing *H. ovis* were aliquoted into each well of the Sensititre plate, incubated at 35C in aerobic conditions, and read at 24 and 48 h.

For biochemical testing on the Vitek2 system, we inoculated 0.45% saline with *H. ovis* to achieve a 0.5 McFarland and entered the cards into the Vitek2 system. The Vitek2 system then made the appropriate dilutions and automatically read them at 15-min intervals until completed, which was 5–8 h, depending on the isolate. We opted to use the Gram-positive ID card because it contains all of the biochemical tests used to identify *H. ovis* in previous studies (Mao et al., 2018). For biochemical testing, we selected the 4 *H. ovis* strains with complete genome assemblies (KG36, KG37, KG104, and KG106) and the 4 *Helcococcus* cryptic strains (KG38, KG95, KG105, KG197). For antimicrobial sensitivity testing, we selected 10 *H. ovis* strains representing each tetracycline resistance gene profile including *tetM* only (KG100, KG106, and KG113), *tetA*/*tetB*(KG36, KG92, KG196), *tetT* only (KG104, KG109, KG120), and none (KG107).

### Pangenome analysis

The *H. ovis* pangenome was constructed using Roary with default parameters and gene clusters were annotated using the BV-BRC Comparative Systems Service (Olson et al., 2023). To identify gene clusters associated with metritis, we used Scoary with default parameters (Brynildsrud et al., 2016). Scoary identifies gene presence or absence variants significantly associated with a trait by performing Fisher’s Exact Tests. It then uses the phylogenetic relations between strains to look for the causal set of genes. Causal genes were defined as those with Bonferroni-corrected *p*-values <0.05.

### Virulome and resistome

Abricate was used to screen all assembled genomes for ARGs using the NCBI AMRFinder and CARD databases^1^ (Jia et al., 2017; Feldgarden et al., 2019). ARGs associated with point mutations were excluded due to a lack of experimental data for the *Helcococcus* genus. Abricate was also used to screen for virulence factors against the VFDB for known plasmids against the PlasmidFinder database (Carattoli et al., 2014; Chen et al., 2016). Virulence factors were further manually searched for using the BV-BRC Comparative Systems Service (Olson et al., 2023).

## Data Availability

The datasets presented in this study can be found in online repositories. The names of the repository/repositories and accession number(s) can be found in the article/Supplementary material.

## Footnotes

^1^ github.com/tseemann/abricate
