# Supplementary material for: Pangenomic and biochemical analyses of Helcococcus ovis reveal widespread tetracycline resistance and a novel bacterial species, Helcococcus bovis
Source: Front Microbiol. 2024 Sep 10;15:1456569. doi: 10.3389/fmicb.2024.1456569 (PMC11420031; doi:10.3389/fmicb.2024.1456569)
Supplement: Supplementary file 5 [file Data_Sheet_5.docx]

| **Isolate:** KG104  **Biochemical Details** | | | |  | | | |  |  |  |  |  |  |  |  |  |  |
| --- | --- | --- | --- | --- | --- | --- | --- | --- | --- | --- | --- | --- | --- | --- | --- | --- | --- |
| 2 | AMY | - | 4 | PIPLC | - | 5 | dXYL | - | 8 | ADH1 | - | 9 | BGAL | - | 11 | AGLU | - |
| 13 | APPA | - | 14 | CDEX | - | 15 | AspA | - | 16 | BGAR | + | 17 | AMAN | - | 19 | PHOS | - |
| 20 | LeuA | - | 23 | ProA | + | 24 | BGURr | - | 25 | AGAL | - | 26 | PyrA | - | 27 | BGUR | - |
| 28 | AlaA | + | 29 | TyrA | (+) | 30 | dSOR | - | 31 | URE | - | 32 | POLYB | - | 37 | dGAL | - |
| 38 | dRIB | - | 39 | lLATk | - | 42 | LAC | - | 44 | NAG | - | 45 | dMAL | - | 46 | BACI | - |
| 47 | NOVO | - | 50 | NC6.5 | - | 52 | dMAN | - | 53 | dMNE | - | 54 | MBdG | - | 56 | PUL | - |
| 57 | dRAF | - | 58 | O129R | - | 59 | SAL | - | 60 | SAC | - | 62 | dTRE | - | 63 | ADH2s | - |
| 64 | OPTO | - |  |  |  |  |  |  |  |  |  |  |  |  |  |  |  |

| **Isolate:**KG105  **Biochemical Details** | | | |  |  |  |  |  |  |  |  |  |  |  |  |  |  |
| --- | --- | --- | --- | --- | --- | --- | --- | --- | --- | --- | --- | --- | --- | --- | --- | --- | --- |
| 2 | AMY | - | 4 | PIPLC | - | 5 | dXYL | - | 8 | ADH1 | - | 9 | BGAL | - | 11 | AGLU | - |
| 13 | APPA | - | 14 | CDEX | - | 15 | AspA | - | 16 | BGAR | - | 17 | AMAN | - | 19 | PHOS | - |
| 20 | LeuA | - | 23 | ProA | + | 24 | BGURr | - | 25 | AGAL | - | 26 | PyrA | - | 27 | BGUR | - |
| 28 | AlaA | + | 29 | TyrA | - | 30 | dSOR | - | 31 | URE | - | 32 | POLYB | - | 37 | dGAL | - |
| 38 | dRIB | - | 39 | lLATk | - | 42 | LAC | - | 44 | NAG | - | 45 | dMAL | - | 46 | BACI | - |
| 47 | NOVO | - | 50 | NC6.5 | - | 52 | dMAN | - | 53 | dMNE | - | 54 | MBdG | - | 56 | PUL | - |
| 57 | dRAF | - | 58 | O129R | - | 59 | SAL | - | 60 | SAC | - | 62 | dTRE | - | 63 | ADH2s | - |
| 64 | OPTO | - |  |  |  |  |  |  |  |  |  |  |  |  |  |  |  |

| **Isolate:** KG106  **Biochemical Details** | | | |  | | |  |  |  |  |  |  |  |  |  |  |  |
| --- | --- | --- | --- | --- | --- | --- | --- | --- | --- | --- | --- | --- | --- | --- | --- | --- | --- |
| 2 | AMY | - | 4 | PIPLC | - | 5 | dXYL | - | 8 | ADH1 | - | 9 | BGAL | - | 11 | AGLU | - |
| 13 | APPA | - | 14 | CDEX | - | 15 | AspA | - | 16 | BGAR | - | 17 | AMAN | - | 19 | PHOS | - |
| 20 | LeuA | (-) | 23 | ProA | + | 24 | BGURr | - | 25 | AGAL | - | 26 | PyrA | - | 27 | BGUR | - |
| 28 | AlaA | + | 29 | TyrA | + | 30 | dSOR | - | 31 | URE | - | 32 | POLYB | - | 37 | dGAL | - |
| 38 | dRIB | - | 39 | lLATk | - | 42 | LAC | - | 44 | NAG | - | 45 | dMAL | - | 46 | BACI | - |
| 47 | NOVO | - | 50 | NC6.5 | - | 52 | dMAN | - | 53 | dMNE | + | 54 | MBdG | - | 56 | PUL | (-) |
| 57 | dRAF | - | 58 | O129R | - | 59 | SAL | - | 60 | SAC | - | 62 | dTRE | - | 63 | ADH2s | - |
| 64 | OPTO | - |  |  |  |  |  |  |  |  |  |  |  |  |  |  |  |

| **Isolate:** KG36  **Biochemical Details** | | | | | |  |  |  |  |  |  |  |  |  |  |  |  |
| --- | --- | --- | --- | --- | --- | --- | --- | --- | --- | --- | --- | --- | --- | --- | --- | --- | --- |
| 2 | AMY | - | 4 | PIPLC | - | 5 | dXYL | - | 8 | ADH1 | - | 9 | BGAL | - | 11 | AGLU | - |
| 13 | APPA | - | 14 | CDEX | - | 15 | AspA | - | 16 | BGAR | (+) | 17 | AMAN | - | 19 | PHOS | - |
| 20 | LeuA | - | 23 | ProA | + | 24 | BGURr | - | 25 | AGAL | - | 26 | PyrA | - | 27 | BGUR | - |
| 28 | AlaA | + | 29 | TyrA | - | 30 | dSOR | - | 31 | URE | - | 32 | POLYB | - | 37 | dGAL | - |
| 38 | dRIB | - | 39 | lLATk | - | 42 | LAC | - | 44 | NAG | - | 45 | dMAL | - | 46 | BACI | - |
| 47 | NOVO | - | 50 | NC6.5 | - | 52 | dMAN | - | 53 | dMNE | + | 54 | MBdG | - | 56 | PUL | - |
| 57 | dRAF | - | 58 | O129R | - | 59 | SAL | - | 60 | SAC | - | 62 | dTRE | - | 63 | ADH2s | - |
| 64 | OPTO | - |  |  |  |  |  |  |  |  |  |  |  |  |  |  |  |

| **Isolate:** KG37  **Biochemical Details** | | | |  | | |  |  |  |  |  |  |  |  |  |  |  |
| --- | --- | --- | --- | --- | --- | --- | --- | --- | --- | --- | --- | --- | --- | --- | --- | --- | --- |
| 2 | AMY | - | 4 | PIPLC | - | 5 | dXYL | - | 8 | ADH1 | - | 9 | BGAL | - | 11 | AGLU | - |
| 13 | APPA | - | 14 | CDEX | - | 15 | AspA | - | 16 | BGAR | - | 17 | AMAN | - | 19 | PHOS | - |
| 20 | LeuA | - | 23 | ProA | + | 24 | BGURr | - | 25 | AGAL | - | 26 | PyrA | - | 27 | BGUR | - |
| 28 | AlaA | + | 29 | TyrA | + | 30 | dSOR | - | 31 | URE | - | 32 | POLYB | - | 37 | dGAL | - |
| 38 | dRIB | - | 39 | lLATk | - | 42 | LAC | - | 44 | NAG | - | 45 | dMAL | (+) | 46 | BACI | - |
| 47 | NOVO | - | 50 | NC6.5 | - | 52 | dMAN | - | 53 | dMNE | + | 54 | MBdG | - | 56 | PUL | - |
| 57 | dRAF | - | 58 | O129R | - | 59 | SAL | - | 60 | SAC | - | 62 | dTRE | - | 63 | ADH2s | - |
| 64 | OPTO | - |  |  |  |  |  |  |  |  |  |  |  |  |  |  |  |

| **Isolate:** KG38  **Biochemical Details** | | | | | |  |  |  |  |  |  |  |  |  |  |  |  |
| --- | --- | --- | --- | --- | --- | --- | --- | --- | --- | --- | --- | --- | --- | --- | --- | --- | --- |
| 2 | AMY | - | 4 | PIPLC | - | 5 | dXYL | - | 8 | ADH1 | - | 9 | BGAL | - | 11 | AGLU | - |
| 13 | APPA | + | 14 | CDEX | - | 15 | AspA | - | 16 | BGAR | - | 17 | AMAN | - | 19 | PHOS | - |
| 20 | LeuA | + | 23 | ProA | + | 24 | BGURr | - | 25 | AGAL | - | 26 | PyrA | - | 27 | BGUR | - |
| 28 | AlaA | + | 29 | TyrA | - | 30 | dSOR | - | 31 | URE | - | 32 | POLYB | - | 37 | dGAL | - |
| 38 | dRIB | - | 39 | lLATk | - | 42 | LAC | - | 44 | NAG | - | 45 | dMAL | - | 46 | BACI | - |
| 47 | NOVO | - | 50 | NC6.5 | - | 52 | dMAN | - | 53 | dMNE | - | 54 | MBdG | - | 56 | PUL | - |
| 57 | dRAF | - | 58 | O129R | - | 59 | SAL | - | 60 | SAC | - | 62 | dTRE | - | 63 | ADH2s | - |
| 64 | OPTO | - |  |  |  |  |  |  |  |  |  |  |  |  |  |  |  |

| **Isolate:** KG95  **Biochemical Details** | | | |  |  |  |  |  |  |  |  |  |  |  |  |  |  |
| --- | --- | --- | --- | --- | --- | --- | --- | --- | --- | --- | --- | --- | --- | --- | --- | --- | --- |
| 2 | AMY | - | 4 | PIPLC | - | 5 | dXYL | - | 8 | ADH1 | - | 9 | BGAL | - | 11 | AGLU | - |
| 13 | APPA | - | 14 | CDEX | - | 15 | AspA | - | 16 | BGAR | - | 17 | AMAN | - | 19 | PHOS | - |
| 20 | LeuA | - | 23 | ProA | + | 24 | BGURr | - | 25 | AGAL | - | 26 | PyrA | - | 27 | BGUR | - |
| 28 | AlaA | + | 29 | TyrA | - | 30 | dSOR | - | 31 | URE | - | 32 | POLYB | - | 37 | dGAL | - |
| 38 | dRIB | - | 39 | lLATk | - | 42 | LAC | - | 44 | NAG | - | 45 | dMAL | - | 46 | BACI | - |
| 47 | NOVO | - | 50 | NC6.5 | - | 52 | dMAN | - | 53 | dMNE | - | 54 | MBdG | - | 56 | PUL | - |
| 57 | dRAF | - | 58 | O129R | - | 59 | SAL | - | 60 | SAC | - | 62 | dTRE | - | 63 | ADH2s | - |
| 64 | OPTO | - |  |  |  |  |  |  |  |  |  |  |  |  |  |  |  |

| **Isolate:** KG197  **Biochemical Details** | | | |  |  |  |  |  |  |  |  |  |  |  |  |  |  |
| --- | --- | --- | --- | --- | --- | --- | --- | --- | --- | --- | --- | --- | --- | --- | --- | --- | --- |
| 2 | AMY | - | 4 | PIPLC | - | 5 | dXYL | - | 8 | ADH1 | - | 9 | BGAL | - | 11 | AGLU | - |
| 13 | APPA | - | 14 | CDEX | - | 15 | AspA | - | 16 | BGAR | - | 17 | AMAN | - | 19 | PHOS | - |
| 20 | LeuA | - | 23 | ProA | + | 24 | BGURr | - | 25 | AGAL | - | 26 | PyrA | - | 27 | BGUR | - |
| 28 | AlaA | + | 29 | TyrA | - | 30 | dSOR | - | 31 | URE | - | 32 | POLYB | - | 37 | dGAL | - |
| 38 | dRIB | - | 39 | lLATk | - | 42 | LAC | - | 44 | NAG | - | 45 | dMAL | - | 46 | BACI | - |
| 47 | NOVO | - | 50 | NC6.5 | - | 52 | dMAN | - | 53 | dMNE | - | 54 | MBdG | - | 56 | PUL | - |
| 57 | dRAF | - | 58 | O129R | - | 59 | SAL | - | 60 | SAC | - | 62 | dTRE | - | 63 | ADH2s | - |
| 64 | OPTO | - |  |  |  |  |  |  |  |  |  |  |  |  |  |  |  |
