# Supplementary material for: Pangenomic and biochemical analyses of Helcococcus ovis reveal widespread tetracycline resistance and a novel bacterial species, Helcococcus bovis
Source: Front Microbiol. 2024 Sep 10;15:1456569. doi: 10.3389/fmicb.2024.1456569 (PMC11420031; doi:10.3389/fmicb.2024.1456569)

**Supplemental File 6.** Approximately-maximum-likelihood phylogenetic tree excluding loci containing elevated densities of base substitutions. Strains highlighted in red contain both high virulence determinants of *H. ovis*. Tree scale represents number of recombination-filtered substitutions across the genome as measured by Gubbins.


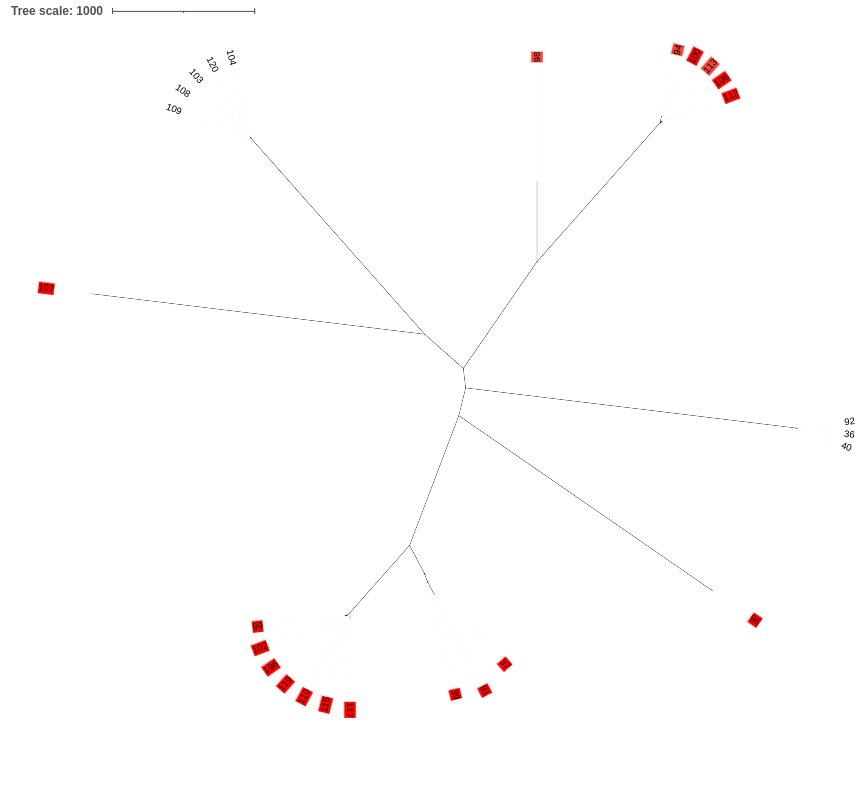

Supplement: Supplementary file 8 [file Data_Sheet_8.docx]
